# Development and feasibility testing of a smart phone based attentive eating intervention

**DOI:** 10.1186/1471-2458-13-639

**Published:** 2013-07-09

**Authors:** Eric Robinson, Suzanne Higgs, Amanda J Daley, Kate Jolly, Deborah Lycett, Amanda Lewis, Paul Aveyard

**Affiliations:** 1 University of Birmingham, Edgbaston, Birmingham B15 2TT, UK; 2 Now at University of Liverpool, Liverpool L69 7ZA, UK; 3 Now at University of Oxford, Oxford OX2 6GG, UK; 4 Now at Coventry University, Coventry CV1 5FB, UK

**Keywords:** Attentive eating, Memory, Attention, Awareness, Food intake, Mobile phone

## Abstract

**Background:**

Attentive eating means eating devoid of distraction and increasing awareness and memory for food being consumed. Encouraging individuals to eat more attentively could help reduce calorie intake, as a strong evidence base suggests that memory and awareness of food being consumed substantially influence energy intake.

**Methods:**

The development and feasibility testing of a smartphone based attentive eating intervention is reported. Informed by models of behavioral change, a smartphone application was developed. Feasibility was tested in twelve overweight and obese volunteers, sampled from university staff. Participants used the application during a four week trial and semi-structured interviews were conducted to assess acceptability and to identify barriers to usage. We also recorded adherence by downloading application usage data from participants’ phones at the end of the trial.

**Results:**

Adherence data indicated that participants used the application regularly. Participants also felt the application was easy to use and lost weight during the trial. Thematic analysis indicated that participants felt that the application raised their awareness of what they were eating. Analysis also indicated barriers to using a smartphone application to change dietary behavior.

**Conclusions:**

An attentive eating based intervention using smartphone technology is feasible and testing of its effectiveness for dietary change and weight loss is warranted.

## Background

The well documented increases in obesity and unhealthy dietary practises mean that there is a significant need for evidence based tools that can help people limit their calorie intake [1,2]. Approaches based around factors known to exert substantial influence on energy intake are likely to be fruitful [3,4]. Moreover, advancements in technology now provide new ways to support people who are trying to reduce their energy intake. For example, smartphone software provides a novel opportunity to aid dietary regulation because the data storage and processing power of handsets enable real-time intervention. An increasing proportion (40% and rising) of both US and UK adults now own smartphones [5,6].

We recently reviewed and meta-analysed experimental studies that examined the influence of awareness and memory on food intake [7]. These studies tested whether manipulating food memory, awareness of eating and distraction whilst eating affect food intake in controlled laboratory settings. The results of this analysis indicated that eating whilst distracted increases concurrent food intake (moderate effect) and to an even greater degree, later food intake (large effect). Moreover, reducing awareness of food consumed was found to increase concurrent food intake and enhancing memory for food consumed was found to decrease later food intake (moderate effect). These results were interpreted within a memory framework, whereby, consistent with earlier findings in the literature [8,9], episodic memories of the ingestive consequences of eating (memory for earlier eating episodes) are suggested to inform decisions regarding future food consumption [7,8]. For example, it has been shown that amnesic patients over-eat [9,10] and that impairing memory of eating results in later over-eating [11]. Thus, awareness of food eaten throughout the day (stored as episodic memories) seems to be important in determining future decisions about how much food to eat. By this account, enhancing episodic memories of eating episodes could be a potential strategy to help people control their food intake [7,8].

Based on the results of the meta-analysis and review we proposed that an intervention aimed at encouraging individuals to eat more ‘attentively’ could help to reduce calorie intake. More specifically, our conceptualization of an attentive eating approach involved encouraging eating devoid of distraction, increasing awareness of food being consumed and prompting of memory recall of food previously consumed prior to eating [7]. Based upon existing theories of the role of memory in the control of energy intake [8,10], we reasoned that these attentive eating behaviors would enhance memory for food eaten and this in turn would reduce over-eating. Here, the development and feasibility testing of an ‘attentive eating’ smartphone application is reported. Our main aims were to test whether overweight and obese participants find an attentive eating smartphone application acceptable to use and use it on a regular basis.

## Method

### Development process

When considering how to design an attentive eating intervention we were guided by the Behaviour Change Wheel (BCW) framework [12]. The main tenet of this framework is the importance of understanding target behaviors in their context and individuals’ existing capability, motivation and opportunity to achieve target behaviors. We therefore considered these factors and how they related to the identified target behaviors, in order to decide what support/tools potential users would need to eat more attentively. A key target behavior of the attentive eating approach is memory recall of earlier food consumed prior to eating. We assumed that individuals would not normally recall earlier eating prior to eating and that in a natural environment there would be a lack of cues to promote recall. Eating whilst distracted may occur because individuals are doing so by habit and/or are not aware of its potentially detrimental effects (a lack of automatic and/or reflective motivation). Individuals may not have the necessary tools or information to increase awareness of food being consumed, as it is commonplace not to store or record eating information after consuming food (a lack of physical opportunity). We identified that a service provision intervention would be best suited, whereby we could design behavioral strategies to increase the likelihood that individuals could complete attentive eating behaviors and learn how to use these strategies.

We also assumed that in order to raise awareness of eating and cue memory recall of food being consumed, the intervention tool would need to be able to store information about eating episodes and then prompt and relay relevant information back to users. We also identified that an intervention that would provide these resources and enable us to educate individuals about the principles and benefits of attentive eating, would help to overcome the earlier discussed existing motivational and physical barriers. Although a paper based tool could achieve some of these considerations, it would be unable to prompt or relay to users relevant stored eating information. Smartphone technology allows for such possibilities. We therefore opted to develop an attentive eating smartphone application. See Table 1 for examples of how a smartphone application was hypothesised to help users overcome barriers to attentive eating.

**Table 1:** BCW strengths of smartphone technology to help users eat more attentively

**Capability**	**Opportunity**	**Motivation**
✓ Smartphone technology can allow for faster recording (embedded camera and touch screen input) and relay (slideshow presentation) of information, in comparison to traditional paper based tools. This strength should make completing target behaviors easier (capability), increase the likelihood that users will have time to complete target behaviors (opportunity) and make behaviors less arduous (motivation).		
✓ Storage and relay of eating episodes in technology increases capability of achieving key target behaviors.	✓ Smartphones are widely used, which should ensure: 1) Easy access to intervention tool (physical opportunity), 2) Socially acceptable tool (social opportunity).	✓ Personalisation of intervention tool to encourage continued use and promote habitual use (automatic motivation).
✓ Automated instructions and guidance to ensure target behaviors are fully completed without error.	✓ Automated reminders to increase number of appropriate opportunities to complete target behavior (physical opportunity).	✓ Storage and presentation of information outlining why the intervention tool will be beneficial (reflective motivation).

### Application

For initial feasibility testing, we designed the attentive eating application for use on the Apple IOS platform (although design features ensured the application could also be developed on other major platforms if later required). The attentive eating mobile phone application was designed with three main components:

1) Snap: Prior to eating/drinking a food or beverage users access the Snap function and select a meal (breakfast, lunch, evening meal, snack, drink, other). This selection loads up a camera view finder and users photograph the food/drink about to be consumed. The user then accepts the photo (or re-takes the shot) and the application relays a short text message reminding users to complete the ‘Most Recent’ function when they have finished their meal.

2) Most Recent: After finishing the meal/drink users access the Most Recent function and the photograph of the recently consumed food/drink is pictured, with information about the meal type and time consumed. With this image on the screen, users select drop down answers to questions about the consumption experience: ‘Did you finish it all?’ ‘How full are you now?’ If users attempt to enter another Snap whilst having outstanding consumption experience questions in the Most Recent section, they are prompted to visit the Most Recent section to complete the outstanding questions.

3) I’ve Been Eating: Prior to deciding what and how much to eat for a consumption episode, users access the I’ve Been Eating Function. This function opens up an interactive chronological slide show of the consumption episodes recorded during that day, starting with the earliest recorded entry (relaying the photograph and all information recorded from the Snap and Most Recent function for each entry on individual screens). A short text message instructs users to ‘remind themselves of what they have been eating’. Users can then navigate forwards and backwards through consumption episodes. If users attempt to move forwards past the most recently recorded consumption episode, they are reminded to eat attentively and to snap their next meal.

**Figure 1:**
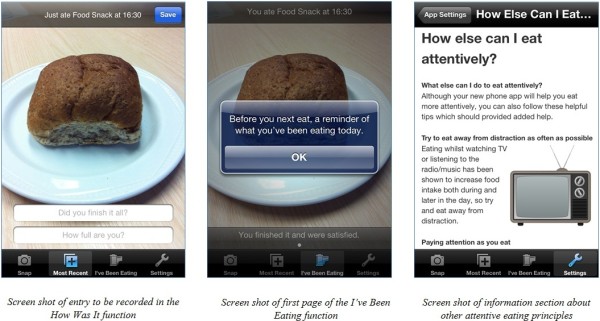
Screen shots of mobile phone application.

Because the three main functions work in a chronological loop, if users attempt to access a function before having completed an earlier function (e.g. trying to enter Snap without having recently used the I’ve Been Eating function), the application provides a text message informing users of this. In a settings function, users are able to personalise the application. Here they are able to enter usual meal times, in order to have automated reminders appear on the main menu of the phone shortly before a usual meal time, to remind them to access the I’ve Been Eating and Snap functions (e.g. users would receive a reminder message shortly before the time they would usually have lunch). Users can also personalise a reminder to instruct them to complete the Most Recent function if the Snap function has been recently used and no post-meal information has been recorded. In the settings function, users can access sections of the application explaining the principles of attentive eating, how these principles might help with energy intake reductions, how to use the application and how to adopt other attentive eating strategies (e.g. eating away from distraction). See Figure 1 for example screen shots of the application.

### Prompting attentive eating

It was reasoned that the behaviors of photographing food/drink and completing questions about consumption episodes should indirectly increase attention paid to and awareness of food/drink being consumed. It was also expected that cueing viewing of photographs and information about foods/drinks consumed that day prior to subsequent consumption episodes would facilitate memory recall of earlier consumption experiences, which would reduce future energy intake [7].

### Feasibility trial

To examine user adherence, acceptance and experiences with the developed attentive eating application, a four week feasibility trial was conducted, in which overweight and obese participants wanting to lose weight used the application.

#### Participants

Participants were staff recruited from the University of Birmingham, UK, during August and September of 2012. Eligibility criteria were: ownership of an iphone (version 2.0 or later), a BMI (body mass index) > 25.0 kg/m^2^, wanting to lose some weight, no history of eating disorders and not being treated with insulin for diabetes mellitus. Twelve overweight (n = 5) and obese (n = 7) participants took part in the four week trial. The sample size was based on published feasibility studies testing mobile phone technology to promote behavioral change [13,14]. At baseline, mean BMI = 32.1 (S.D = 5.3), mean weight = 96.3 kg (S.D = 19.0). Mean (S.D = 12.4) age was 41.7 years, with seven females and five males participating. Participants were compensated with £30 sterling for their time (equivalent of $47 US dollars). The trial protocol was approved by the University of Birmingham Ethics Review Board.

#### Procedure

A researcher screened participants over the telephone and arranged a convenient date and time to meet. In this first session, a researcher explained the nature of the trial and participants were provided with an information sheet. To confirm that the self-reported BMI collected during telephone screening was accurate, the researcher measured height (stadiometer) and weight (Tanita TBF-300MA weight scale). The researcher explained the concept of attentive eating, the nature of the mobile phone application and why its use may be beneficial for reducing food intake and managing hunger. Participants were asked whether they were on medication (two were taking non-appetite related drugs) or following a structured weight loss plan (no participants were). Participants gave informed consent and completed a questionnaire measuring demographics. The application was then installed on the participant’s phone. The researcher demonstrated the main functions of the application. For use in the feasibility trial, a database was included with the application that recorded a log of button pressing. This allowed for examination of application usage and participants were informed that in the final session the researcher would download this database from their phone. The design of the application ensured that users would not be able to use their phone to make alterations to the logged data. The researcher then arranged a follow up session to take place two to three days later.

During the second session, the researcher asked participants if they had been using the application, whether there were any problems with the application and if they were happy to continue. A final follow up session was arranged approximately 28 days from the first session. In the final session, the researcher first conducted and recorded a semi-structured interview with the participant. Open ended questions were based around participants’ impressions and experiences of using the application. This interview was recorded and lasted approximately 15 minutes. Once the interview had been completed, participants next completed a self-report questionnaire, consisting of five-point Likert scaled questions (strongly agree to strongly disagree) designed to measure ease of use (‘by the end of four weeks I found the app easy to use’), convenience and integration into daily routine (‘by the end of the four weeks I found the app easy to use’/had fitted into my routine’) and future intentions to use the application (‘I intend to use the app in the future’). The button pressing log was then downloaded from the participant’s phone, before weight was re-measured and participants were paid, thanked and debriefed.

#### Measures of interest

Our primary measures of interest were frequency of usage (measured via the button pressing log), qualitative accounts of the effects of the application and factors affecting usage, as well as self-reported acceptance of the application (as measured in the questionnaire). We also examined changes in body weight.

## Results

### Application usage data

Participants were enrolled in the trial for a mean average of 27.5 days (S.D = 2.8). Participants accessed the application 5.7 times a day on average (S.D = 2.5). The mean number of eating and drinking episodes recorded each day was 2.7 episodes (S.D = 1.5). The mean number of episodes entered each day for the different meal types for breakfast was 0.7, for lunch was 0.5, for an evening meal was 0.6, for a snack was 0.6 and for a drink was 0.4. Of these entries, 97.9% (S.D = 2.2%) contained complete information, in that entries were made after having accessed the I’ve Been Eating function and all information requested in the Most Recent function was provided. The mean time between entering a photograph in the Snap function and completing the Most Recent function was 126 minutes (S.D = 164). This long time gap appeared to be caused by a few participants leaving large time gaps between entering the Snap and Most Recent function. Thus, some participants did not use the mobile phone application as was intended. However, for the majority of the participants, the time gap was approximately one hour or less. During the trial, participants personalised usual meal times (mean number of meals personalised = 2.4, S.D = 1.9). Thus, users tended to alter the default settings of the application so that they would receive a reminder about using the application shortly before some of their usual meal times. The application was accessed M = 12.8% (S.D = 8.5) of the time via a reminder message. This indicates a modest benefit of including the reminder settings, as in these cases users were directly accessing the application through clicking on a reminder notification. These descriptive data suggest that participants engaged with the application during the trial, and that they used the application regularly to access and complete the main functions.

### Self-reported acceptance

Descriptive statistics showed that users found the application easy to use (M = 4.7/5, S.D = 0.5) and intended to use it in the future (M = 3.8/5, S.D = 1.2). Convenience and integration into daily routine were both rated around the midpoint of the scale (M = 3.3/5, S.D = 1.1 and M = 2.9/5, S.D = 1.4). These descriptive data suggest that the application was easy to use and users would consider continued use, but users may require further support for use of the application to become a convenient or habitual day-to-day behavior.

### Qualitative analysis

Thematic analysis of transcripts of the recorded interviews was used [15], in order to identify themes relating to application usage and behavioral effects/experiences of using the application.

Three themes relating to application usage emerged.

Theme 1: The role of *Automaticity* appeared to be an important factor influencing usage. Users reported that a significant challenge to regular use was getting into the habit of using the application and spontaneously remembering to use the application before and after meal times. See Table 2 theme 1.

**Table 2:** Thematic analysis results: themes relating to usage and effects on behavior

		
**Theme 1: Automaticity**	**Theme 2: Mobile Phone accessibility**	**Theme 3: Social Contexts**
EXTRACT 1	EXTRACT 4	EXTRACT 7
*Pps 11: If you’re having a really busy day then you know it could slip out of your mind.*	*Pps 2: The times when it was tedious was when I didn’t have my phone on me for some reason.*	*Pps 2: If I was with people they would just say why are you taking a picture of your dinner and I’d just explain to them and that was fine.*
EXTRACT 2	EXTRACT 5	EXTRACT 8
*R: Is there anything you think could be done to improve the app?* *Pps 1: How you can use the app to force, not force habit, but enhance it as a habit.*	*Pps 5: Weekends tend to be a bit of a pain, the phone is in one room, you’re in another, or up the garden somewhere.*	*Pps 11: When you’re at work where you don’t want to be seen to be fiddling with your phone all the time, because obviously you’re there to work, so that’s one negative I suppose, but then that’s just a personal thing.*
EXTRACT 3	EXTRACT 6	EXTRACT 9
*R: So would you be motivated to continue using it then?*	*Pps 10: Because it’s on the phone, you know I have my phone with me all the time anyway, so it’s very convenient.*	*Pps 4: I didn’t get any strange looks when I was in Starbucks.*
*Pps 10: Yeah, I would still try and it’s about changing habits I think.*		
**Theme 4: Raised awareness**	**Theme 5: Changing other behaviors**	
EXTRACT 10	EXTRACT 13	
*Pps 4: I found it very useful, it made me much more aware of what I was eating and it’s actually made me modify what I’m eating as well.*	*Pps 1: I’m thinking about doing it before using the app, so I’m having this therefore I’m going to be recording it, therefore do I need the biscuit?*	
EXTRACT 11	EXTRACT 14	
*Pps 3: There have been occasions at work where I’ve thought I fancy something to eat and I would have a look at what I’d eaten, then I think ‘you’ve had enough, you can wait till lunch’.*	*Pps 6: The stuff that I read on it, you know the ideas about not eating in front of the TV and all that stuff, that stuff makes sense and you know I’ve changed how I do that.*	
EXTRACT 12		
*Pps 12: But even now it has made me think more about food more, so I do think I’ve already eaten that and eaten that today.*		

Theme 2: *Mobile phone accessibility* was identified as both a barrier and aid to continued usage. Users reported that adherence was impeded by day-to-day activities that resulted in them not having their phone (see Table 2 extracts 4 and 5). However, for some users, regular use of their mobile phone made the intervention tool convenient to use (see Table 2 extract 6).

Theme 3: Some participants reported that in a minority of *social contexts*, they felt that using their mobile phone was inappropriate and this made them less likely to use the application, (e.g. whilst at work – see Table 2 extract 8). However, amongst other participants there was a feeling that using their mobile phone was socially acceptable in the context (see Table 2 extract 7 and 9).

Two themes relating to behavioral effects of using the application emerged.

Theme 4: Participants felt that application usage *raised awareness* of what they had been eating and at times this information resulted in changes to decision making regarding future eating. See Table 2 theme 4.

Theme 5: Participants also reported that using the application produced *other changes to behavior*. For example, the mere presence of the application on their mobile phone and the act of having to photograph and record foods was perceived to make participants think more carefully about what they should be eating (see Table 2 extract 13). This is different to the Theme 4 as it suggests that the application may have influenced behavior through mechanisms (such as guilt or self-monitoring) other than memory for food intake.

Participants also reported that possessing the application resulted in them adopting other attentive eating principles, such as stopping eating in front of the television (see Table 2 extract 14), which again does not relate directly to the main functions of the application.

### Weight change

Mean weight loss was 1.5 kg (S.D = 2.8). 6/12 participants lost 1 kg or more, four lost between 0-1kg and the remaining two participants gained between 0.1 and 0.4 kg.

## Discussion

We developed and tested the feasibility of a smartphone application designed to help people eat more attentively. Adherence data suggested that overweight and obese participants in this four week trial used the application regularly, personalised the application based on their daily routine and were able to use the three main functions of the application (Snap, Most Recent and I’ve Been Eating). Participants reported that they found the application easy to use and would consider using the application in the future. Qualitative analysis indicated that participants felt that using the application raised their awareness of their dietary practises and that they could make use of this information to inform eating decision making. On average, participants lost 1.5 kg weight by the end of the four week trial.

The results of the feasibility trial are promising, as there was evidence of good adherence to the intervention. Accounts of the application being easy to use, raising awareness of eating and weight loss achieved suggest this approach could be fruitful in future weight loss interventions. The 1.5 kg average weight loss observed is similar to a recent more intensive two month trial that investigated the impact of dietary/exercise advice and habit formation on weight loss [16]. Given that our trial was a very brief intervention with little contact time and no nutritional advice or support, this is a promising finding. The clinical significance of the amount of weight lost requires further investigation. For example, if this level of weight loss was achieved at a constant rate in subsequent months, this intervention would be clinically significant. However, as the main aims of the present research were to assess initial feasibility, we are unable to draw conclusions about clinical significance. Moreover, as the sample size in the feasibility study was small, the conclusions made are tentative. Nevertheless, a randomized controlled trial testing proof of principle for an attentive eating intervention on weight loss is warranted, so that it will be possible to examine any potential benefits to weight loss over a longer period of time.

Potential barriers to continued use of this smartphone application were also identified. Qualitative analysis indicated that consideration of social contexts that could cause negative or critical judgements from others for using an eating/weight loss application may be important. Given that stigma is attached to obesity and perceived ‘over-eating’ [17,18], there may be social contexts that make users feel uncomfortable about using the application. Given that a sizeable proportion of meals are likely to occur in social settings, alternative attentive eating strategies (e.g. a structured mental recall of food eaten) may need to be developed for use in such scenarios. Qualitative analyses also suggested that additional practices that encourage attentive eating to become ‘automatic’ or habitual may be needed. By the end of this four week trial participants were not sure if the application had fitted into their daily routine. This is perhaps not surprising as eating behavior habit formation can take upwards of twelve weeks [19]. Habit formation is important if practitioners are to help patients achieve long-term change to usual dietary practises [20].

There are limitations to the present work. As this was a feasibility trial there was no control group to allow for testing of effectiveness. In addition, as the main aim was to investigate feasibility and user acceptance we did not include a direct behavioral measure to detect whether participants’ memory and awareness of eating episodes had improved. Future research will be better suited to answering these questions. However, it should be noted that transcripts of the interviews suggested that participants felt their awareness had improved. How this kind of intervention could produce long term changes to eating behavior also needs consideration. Although adherence data was promising, whether users would continue over longer periods of time will need to be examined. This is important, as long term maintenance of changes to the diet and weight can be hard to achieve [21,22]. It may be possible to use this mobile phone application to train individuals to get into the habit of eating more attentively and then gradually remove of use of the application. As participants were University staff, it could also be argued that their appreciation of research could be higher than the average person, which may have increased adherence.

## Conclusion

In conclusion, the present work introduces an attentive eating approach that is supported by theoretical models of the role of memory on energy intake regulation and aimed at reducing dietary intake and promoting weight loss. The results suggest that a simple smartphone based intervention based on these principles is feasible and could promote healthier dietary practises.

## Competing interests

All authors declare no competing interests.

## Authors’ contributions

All authors were responsible for the design and development of the intervention. ER, PA and SH conceived the feasibility study, participated in its design and co-ordination. ER conducted the research and analysed the data. ER, PA and SH were responsible for the first drafting of the manuscript and all authors approved the final manuscript.

## Pre-publication history

The pre-publication history for this paper can be accessed here:

http://www.biomedcentral.com/1471-2458/13/639/prepub
